# Deaf legal theory: challenging the law’s hearing bias

**DOI:** 10.1093/jdsade/enaf010

**Published:** 2025-02-17

**Authors:** Rob Wilks

## Abstract

Bryan and Emery introduced a new concept in legal jurisprudence through which a critical examination of how the law deals with deaf people can be undertaken: deaf legal theory (DLT). They define it as “how the law seeks to frame Deaf people” and argue that legal systems should be reoriented to recognise and accommodate the unique perspectives and experiences of deaf people. Current legal systems are biased in favour of hearing people and these bias disadvantage deaf people in a variety of ways, including in their access to justice, employment, and education. The aim of this article is to advance Bryan and Emery’s DLT by expounding its main arguments, situating it within its jurisprudential home of critical legal studies, considering the justification for its existence and providing a framework to apply it. The concept was introduced not within legal discourse but within Deaf Studies discourse and is therefore not yet widely known in legal scholarship. This article aims to bridge the gap between the two disciplines and firmly establish DLT as a legal theory in jurisprudence following which it can be applied to various legal subjects of intellectual enquiry.

Jurisprudence, in its simplest form, is the philosophy of law, and allows for a critique or evaluation of the law by which people are expected to abide, and it is made up of several legal theories and approaches, all of which attempt to explain the law and legal institutions in their historical, philosophical and political contexts. The study of jurisprudence allows researchers to analyse and think critically about the law utilising tools from particular disciplines (Bix, 2015; Ratnapala, 2017) and helps scholars and society in general to better comprehend the law, legal systems, and legal reasoning at national, supranational or intergovernmental level expressive of political and economic power and has “generous frontiers” (Wacks, 2015, p. 5). In short, jurisprudence and legal theory promote a fundamental understanding of the law, although they do not always provide answers to what the law is or what it is meant to do, but can instead offer pointers, clues, and insights (Freeman, 2014).

Matsuda (1987) reminds us that those who have experienced discrimination speak with a special voice to which we should listen, necessitating a “looking to the bottom” approach from the perspective of deaf people and Deaf communities. Note that throughout this article, reference is made to “deaf” or “Deaf.” For clarification, “deaf” is used to describe all kinds of deaf persons, and “Deaf” is used to refer to sociocultural entities or established theoretical concepts such as “Deaf community.” It is past time for attention to be paid to deaf communities and their experiences due to the various social, political and economic forces at work. It is imperative to acknowledge the dearth of extant literature or research pertaining to legal theory that specifically addresses the rights and concerns of deaf individuals. There is a paucity of comprehensive research or scholarly publications that have investigated this important dimension of legal discourse. In response, I am advancing a legal theory known as deaf legal theory (DLT), which represents a novel concept in jurisprudence through which a critical examination of how the law deals with deaf people can be undertaken. DLT was devised by Bryan and Emery (2014), who define it at its simplest as “how the law seeks to frame Deaf people.” This article seeks to expand Bryan and Emery’s DLT by expounding its main arguments, situating it within its jurisprudential home of critical legal studies (CLS), and explaining it by unpacking its meanings and exploring how it can be applied. The aim is to firmly establish DLT as a legal theory in jurisprudence following which it can be applied to various legal subjects of intellectual enquiry.

It is necessary to bridge the gap between legal and Deaf studies and firmly establish DLT as a legal theory in jurisprudence simply because the law is audist by default. Audism is a term coined by Humphries (1977), from the Latin *audire* (to hear), and he refers to it as “the notion that one is superior based on one’s ability to hear or behave in the manner of one who hears.” Eckert and Rowley (2013) define it as treating people differently based on how well they can hear and communicate due to their own or society’s assumptions and prejudices, and Bauman (2004) posits that audism involves the devaluation of deaf people due to stereotypes that paint them as incompetent and intellectually inferior. In short, audism is about hearing people thinking (consciously or subconsciously) that they are superior to deaf people, and treating deaf people according to these thoughts, assumptions, prejudices and stereotypes.

Audism also extends beyond individual attitudes. It operates as a systemic and cultural framework, deeply embedded within social and institutional structures (Lane, 1992; O’Connell, 2022). Bauman (2004) refers to this as a “system of advantage” for hearing people, and Eckert and Rowley (2013) describe audism as prioritising hearing ability over deafness. This applies to the law itself: audism occurs when the law frames deaf people through a hearing lens, rather than on their own terms as deaf people, by assuming that all its subjects are hearing. Indeed, even very many well-intentioned protective laws may undermine the rights of deaf people (Bagenstos, 2004). It is also argued that the law—however well-intentioned it may be—has a constitutive role in the labelling of deaf people as disabled and ignoring Deaf culture and the importance of sign language to the Deaf community, what I refer to as the Deaf Legal Dilemma due to the Deaf-disabled and language-minority dichotomy (Wilks, 2019; Wilks, 2022). The law generally recognises Deaf-disabled rights and affords protections and benefits if deaf people accept that classification. Language-minority rights do also exist, usually in the form of sign language recognition law, but generally do not proffer the same level of protection or benefits as Deaf-disabled rights. Ferri et al. (2024) expand Wilks’ taxonomy of disability and language minority rights to a third group, cultural-linguistic rights, which sits somewhere between the two. The purpose of DLT is to expose this audism. Only by identifying and understanding it can we take the necessary steps to address it.

Throughout the article, I use the United Kingdom (UK) as a case study to illustrate the application of DLT and identify gaps in UK law. This approach leverages my familiarity with UK law while demonstrating how DLT provides a universal framework that can be applied to legal systems and areas of law globally. In the UK, deaf people are more likely to be underemployed or unemployed due to the challenges they face to gain and remain in employment, and they face additional attitudinal and practical barriers (Wilks, 2019). Totaljobs (2016) found that discrimination plays a large part in the working lives of deaf people, and the attitudes of employers and colleagues can prevent deaf people from fulfilling their potential, and often lead to them feeling isolated at work. Linking this discrimination to audism, O’Connell (2022) argues that stigma and stereotypes, institutional audism, glass ceiling discrimination, and internalised audism all play a part, due to stereotyped characterisations of deaf people as disabled people rather than as a language minority.

These discrepancies in employment are further compounded by deaf individuals’ educational experiences, as they frequently leave compulsory education with a reading level of an 8-year-old (Rowley, 2023) and there are persistent achievement gaps between deaf and hearing children attributed to expecting hearing aids or cochlear implants to have excellent effects when outcomes vary, low expectations and poor access to learning in school classrooms, and language deprivation (Wilks & O’Neill, 2024). Furthermore, the Deaf community generally experiences inferior health outcomes due to limited access to healthcare services (Emond et al., 2015), and are twice as likely to grapple with mental health challenges compared to their hearing counterparts (Terry et al., 2021). There is also a shortage of British Sign Language (BSL) interpreters and translators (Department for Work and Pensions, 2017). Meanwhile, inadequate language and communication access during childhood results in a higher risk of developing chronic diseases and mental health disorders in adulthood (Kushalnagar et al., 2020).

Equality law is generally failing to achieve its aims in eliminating inequalities and discrimination against deaf people (Wilks, 2019), compounding these issues. As national sign languages ensure deaf people’s optimal mental, physical, and social health across the lifespan (Snoddon et al., 2022), and optimises brain development and prevents language deprivation (Snoddon & Paul, 2020), it falls upon language-minority rights to provide redress. For example, Paul and Snoddon (2017) argue for deaf children’s right to sign language to be recognised in the Canadian Charter of Rights and Freedoms to meet their biological and linguistic needs, and Wilks (2022; 2019) argues that sign language recognition is the solution to the inadequacies of equality law and has the potential to achieve transformative equality for deaf people.

For ease of navigation, this article is arranged as follows. Following the introduction, the article begins with a detailed description of what DLT is. Next, I explain how DLT fits within existing jurisprudence, and the various critical approaches to law. I then set out the eight tenets that form the DLT model, giving researchers the wherewithal to examine legal systems or areas of law through a DLT lens. The paper then reaches its conclusion and discusses future directions for DLT.

## What is deaf legal theory?

Bryan and Emery's (2014) exploration of DLT delves into the ways in which the law frames the status of deaf individuals within society. This discussion emphasises the need for a paradigm shift in legal perspectives by introducing the concept of Deaf jurisprudence—the law relating to Deaf issues—highlighting its absence in legal discourse. Their primary argument is that the law plays a pivotal role in perpetuating the subordinate status of deaf people, citing instances such as restrictions on certain occupations and the framing of Deaf issues as matters of “special educational needs.” To reconstruct this relationship, the authors propose the emergence of Deaf Gain to challenge prevailing norms, and to give due consideration to deaf people’s perspectives.

The concept of Deaf Gain is a powerful framework for understanding the positive contributions of deaf individuals to jurisprudence. It refers to the unique cognitive, creative, and cultural gains manifested through deaf ways of being in the world (Bauman & Murray, 2014). There are more precise aspects to the nature of this gain, which include DEAF-BENEFIT, DEAF-CONTRIBUTE and DEAF-AHEAD. DEAF-CONTRIBUTE is the most relevant and refers to the contributions of deaf individuals, communities, and their languages to humanity, that is, to a more robust biocultural diversity, such as the new perspectives on human nature that have arisen from the study of signed languages (Bauman & Murray, 2014). If life is improved for deaf people, life is improved for the whole of society as the law considers and includes their unique perspectives and experiences, recognising their value and contribution. After all, deaf people have something to teach, and challenge mainstream discourses that often frame them in a negative light. By emphasising positive representations and employing Deafhood-related terms, Bryan and Emery (2014) argue for a paradigm shift to challenge existing assumptions and promote a more inclusive legal landscape.

Bryan and Emery (2014) raise pertinent questions about societal viewpoints, media representation, and the exclusion of Deaf voices from public discourse, exposing the Human Fertilisation and Embryology Act (2008). This legislation embodies significant ethical and legal challenges, as section 14(4)(9) explicitly prohibits the selection of embryos likely to result in a child with a “serious disability,” including being deaf, reinforcing narratives that position deaf lives as undesirable and echoing historical eugenics movements that sought to exert control over reproduction. They challenge assumptions that underpin legal frameworks, emphasising the potential bias in favour of majority perspectives. This exclusion, they argue, contributes to a “hearing-subjective” society, limiting the understanding of Deaf experiences and imposing a “cultural order” on deaf people, with the law being the ultimate formal expression of this order. In fact, the law affords “privilege” to deaf people who fit in within the expectations of the dominant hearing society—the “hearing construct”—of who they should be.

For example, the law provides for the education of deaf children in mainstream settings, allows deaf people to qualify for disability-related benefits, provides funding for adjustments in the workplace, and public funding through the national health service for medical interventions such as cochlear implants and hearing aids (Bryan & Emery, 2014). Those who adopt a liberal or accepting stance on this matter are often referred to as “assimilationists,” as their arguments predominantly revolve around achieving equal opportunities within current frameworks, rather than challenging established standards, rules, or structures (Chamallas, 2012). However, when deaf people challenge the status quo, they are often framed as being reactionary, and this has an adverse effect whereby deaf communities position themselves defensively with an almost “underground” mentality which can have a negative impact on the Deaf state of being (Ladd, 2003).

To find a way forward from the current state of affairs, Bryan and Emery (2014) advocate that we need to move on from equality theory to recognise and celebrate difference in terms of biology and identity, and be aware of the “economics of deafness,” responsibility and the “cost to society” arguments and consider the role of intersectionality in developing Deaf jurisprudence. Firstly, they posit that the current model used to govern deaf people’s relationship with the law is that of equality theory, and that the approach is one of assimilation (Bryan & Emery, 2014). There is an implication that we need to move away from equality theory in order to develop Deaf jurisprudence, but I remain steadfast in my commitment to equality law as a way forward (Wilks, 2019). To undertake the challenging task of examining the relevant theoretical perspectives of equality law as they relate to deaf people, I devised a precept methodology. Formal, substantive and transformative equality are the three precepts of equality law. The concepts of equality relevant to deaf people can then be grouped into each precept, as each concept will contain features that align naturally to at least one of them. Thus, the precept of formal equality includes the concepts of equal treatment and equality of opportunity, whereas substantive equality comprises respect for equal worth, dignity and identity and equality of results, and finally, transformative equality which can be linked to social inclusion, challenging oppression and seeking full participation. I conclude that while formal equality has limited use and substantive equality can only ever be temporary due to deaf people’s need for recurrent adjustments, transformative equality has the potential to achieve equality for deaf people. However, due to its scarcity in anti- and non-discrimination law, it is a yet underdeveloped method of achieving equality for deaf people and therefore merits further consideration (Wilks, 2019).

The second model referred to by Bryan and Emery (2014) is that of recognising and celebrating difference. They critique the tendency to label deaf individuals as “abnormal” as seen by definitions of disability in anti-discrimination law which often refer to individuals’ “impairment,”^1^ thus labelling deaf people as inferior or damaged goods. Instead, they argue for a model that values diversity rather than reinforcing inferiority. By exploring the biological, identity, and economic dimensions of Deafness, they emphasise the need for a more nuanced understanding that goes beyond prevailing stereotypes. Rather than pouring resources into the dominant group’s narrative, Bryan and Emery (2014) advocate for acknowledging the potential advantages in deaf people’s bodies which suggest a unique sensory advantage for deaf individuals. Additionally, they draw on critical race theory to emphasise that Deaf identity, like race, is not solely biological but a crucial component of human diversity, and that deaf people are an ethnic group with linguistic and cultural biodiversity. The concept of deaf people as an ethnic group is widely debated. While this framing emphasises shared cultural and linguistic practices, critics like García-Fernández (2014) caution against oversimplifying deaf identity. Intersectional analyses, such as those by Moges (2020) and Friedner (2017), further argue that framing deaf communities as a singular ethnic group risks neglecting the impact of race, class, and colonial histories on deaf experiences. Nevertheless, this perspective affords leeway to recognise and protect deaf people’s language and human rights, moving beyond a deficit-oriented approach to celebrate the richness of their unique experiences and contributions.

In terms of the third model, “economics of deafness,” being deaf is often seen as costly, contributing to the economic subordinate status of deaf individuals. This reinforces notions of dependency and privilege, bringing attention to welfare issues. Bryan and Emery (2014) further critique approaches that focus solely on calculating the expense of deafness to society, arguing that these perspectives assume these costs exclusively benefit deaf individuals. This calls for a more nuanced understanding of the economic implications of deafness within a broader societal context. The majority view is that allowing deaf children to be born through in vitro fertilisation would incur a high cost to society in terms of burden on the state, but Bryan and Emery (2014) posit that in fact, society would be deprived of DEAF-CONTRIBUTE if there were fewer deaf children in deaf families. More deaf children born to deaf parents will ensure the transmission of Deaf culture across generations, preventing societal fragmentation and preserving a rich learning environment. Finally, Bryan and Emery (2014) suggest that intersectional analysis is essential in understanding Deaf experiences within legal contexts, emphasising its potential to challenge existing perspectives and enrich social justice discussions (see Friedner, 2017; Moges, 2020). By building a Deaf jurisprudence based on the principles of Deaf Gain, they argue for a framework where being Deaf is neither better nor worse but merely different.

## Situating deaf legal theory

The principal reasons for studying jurisprudence are also reasons for applying a DLT lens. They are predominantly intellectual. Simmonds (2018) asserts that a comprehensive understanding of the law necessitates a study of jurisprudence, wherein the fundamental inquiries about the nature and function of law are addressed, by an examination of the law’s involvement in shaping hierarchies and their connection to the exclusion or marginalisation of disabled individuals (Kanter, 2011), the focus should be on the exclusionary aspects and the potential for inclusion, as well as the concept of Deafness (Weber, 2012). Neglecting this endeavour would leave legal practitioners ignorant of the applicable ideas and frameworks essential for addressing the needs of the Deaf community (Simmonds, 2018).

### Critical legal studies

There is considerable jurisprudence regarding various critical approaches to law through which a critical examination of law can be made, and attempts have been made to do so by legal positivists, natural lawyers, legal realists and through CLS. For present purposes, it is submitted that DLT is firmly entrenched in CLS discourse. Goodrich et al. (1994, p. 6) succinctly describe CLS as “a study both of the failures, of the injustices, the exclusions and the inequalities of the legal tradition and an examination of the future of law.” The primary CLS scholars of the 1980s^2^ acknowledge that there are three unifying “critical” elements common to CLS and its offshoots which include: the historicisation of the problem being examined, how the law can be used to resist modes of domination and subordination by identifying what is immovable (necessary) or what could be different (contingent) and considering the language of “surface” and “depth”; and how the law operates in and through “culture.”

The first critical element is that of historicisation. The historicism of legal scholarship in relation to deaf people include deaf people’s experiences in the Old Bailey in the eighteenth and nineteenth centuries (Woll & Stone, 2008), the capacity of deaf persons in the Imperial Court of Justice in Leipzig between 1880 and 1900 (Enescu & Werner, 2016), and an examination of deaf people’s capacity, crime and punishment and role as witness and victim in eighteenth-century England (Wilks, 2021). However, the question as to whether there is such a thing as “too much history” (Wilson, 2017), that is, focusing primarily on the legal historicisation of deaf people, has been discussed. Critics raise several concerns such as its isolation from broader societal contexts, the conservative use of history and the overlooking of marginalised groups in legal history research (Baker, 2000; Gordon, 1984; Ibbetson, 2001; Wilson, 2017), and theories of how law relates to society and to history can minimise such threats (Gordon, 1981). Such theorisation can consist of old or new concepts and theories to help make sense of past events. For instance, Wilks (2019) examines why equality law is failing deaf people by utilising equality theory to identify relevant concepts of equality, and McEvoy (2021) investigates the lived experiences of Deaf Irish Sign Language users who have interacted with the criminal justice system utilising identity and justice theory.

In relation to the second critical element, Hunt (1986) explains that critical scholars see the law as a “significant constituent in the complex set of processes which reproduces the experience and reality of human subordination and domination” (p. 43). This is pertinent in the case of deaf communities, as Bryan and Emery (2014) argue that deaf perspectives are often not given due consideration as they do not have their own autonomous space within the larger, hearing-dominated democratic society. The response is to consider how the law can be used by deaf people to resist subordination and domination, and this too requires an historiographical approach to understand the role structural forces play in the problem being examined. To do so, an examination of what is immovable (necessary) or different (contingent) is generally required. Painter (2021, p. 48) explains that the initial focus was “timeless truths, necessity, and structural explanations,” often associated with legal formalism, but due to CLS, this shifted to contingency, and is now returning to “empiricist, objective, and event-narrating methods.” A detailed discussion of the definitions of “necessary” and “contingent” is beyond the scope of this article, but can be summarised thus: “necessary” speaks to a course of events that are, in a way, pre-determined, as “the past shapes the possibilities of the present,” and “contingent” suggests that the course of events is not fixed, and “is very often strange and unexpected” (Marks, 2009, p. 1).

Venzke (2021) explains how such an approach could work in practice: first of all, by contextualising the law, all the while resisting the inevitability of that context, and then considering what other paths or outcomes were possible within and against that context. These paths or outcomes can formulate a strategy and tactics to effect any change (Knox, 2010). To illustrate, historicisation could be examining the impact of the Second International Congress on Education of the Deaf in Milan in 1880, at which a declaration was made that oralism was to be the method of education for deaf children and a resolution passed banning the use of sign language in schools. Before embarking on a “what if” study of this topic, that is, what would have happened if the use of sign language in schools had not been banned, the context that led to the declaration and resolution would first need to be examined (such as Viera-Machado & Rodrigues, 2022). A word of warning however: just because a contingency or contingencies are identified, does not mean that things will change (Marks, 2009).

In terms of “surface” and “depth,” this where the themes of subordination and domination come into play. The notion of the “surface” is often linked to that which is superficial and misleading, pertaining to what can be observed without thorough analysis and, by implication, would ultimately prove to be untrue upon careful examination (Best & Marcus, 2009), whereas “depth” requires a higher degree of thoroughness and examining a wider array of choices, providing comprehensive rebuttals, and making nuanced differentiations (Ben-Zvi, 2019). Therefore, on the “surface” of law, everything appears to be flawless, whereby the judiciary, lawyers and politicians are all committed to one overarching aim: to be democratic and responsive to the demands of the people, whereas in the “depths” lie misogyny, prejudice, discrimination, coloniality and capitalism. The purpose of this exercise is to bring the relationship between knowledge, structure and agency (that is, the individuals who build the systems (Gordon, 1984)) to the surface to challenge social structures that are based “upon a skewed perspective of reality” (Eslava, 2020).

The final critical element of CLS is understanding how the law operates in and through “culture,” whereby the law is regarded a form of literature (Douzinas & Gearey, 2005). Cultural legal studies is not a study of culture or cultures but draws on the techniques, approaches and methods drawn from cultural studies either explicitly or by association (Leiboff & Sharp, 2016), such as literature, art, photographs, theatre, and cinema (Crawley, 2016). Notwithstanding the ongoing debate about whether legal texts constitute “literature” (see Douzinas & Gearey, 2005; Singh, 2019), a recent example is Sousa’s (2023) exploration of Deaf culture in legal texts at supranational and national level, concluding that there is a variance in definitions, and a scarcity of explicit designations.

## The plurality of critical legal studies

Having considered the three critical elements of CLS and their relevance to DLT and deaf people, let us move on. CLS discourse has shifted to recognise a multitude of critical perspectives which all contribute to the plurality of CLS (Parsley, 2018). These perspectives have contributed to the emergence of feminist legal theory, critical race theory and critical disability theory, all of which emerged around the same time to address the social construction of difference to justify oppression (Liasidou, 2014; Rocco, 2005), with the aim of exposing and dismantling the systemic inequities and barriers facing marginalised groups. It is possible to intersect these identities, and Crenshaw's (1991, 1989) concept of intersectionality helps us understand how multiple forms of inequality or disadvantage can intersect and create unique obstacles that are often overlooked by conventional approaches. This understanding is useful for addressing the broader issues experienced by deaf individuals, as they may not necessarily experience audism in isolation. The deaf identity may intersect with other identities, such as gender, race, sexuality, and religion, compounding the issues and creating additional obstacles.

The three critical theories help develop a deeper understanding of oppression and pathways to remedy discrimination. Within this context, Matsuda (1987) describes CLS as using “a sharp knife to cut through existing assumptions about law” (p. 350). These critical legal perspectives collectively reject the idea that the law is impartial. In essence, they question the neutrality and value-free nature of the law and those who create it (Patterson & White, 1999). The law is filled with conflicting values and the outcomes of political struggles, making it difficult for the judiciary, who interpret the law, to fully comprehend it and despite their efforts to resolve inconsistencies and downplay conflicting values, these contradictions eventually become apparent (Howarth, 1992) as their judgments tend to reinforce the status quo rather than effect change. The work of critical legal theorists enables us to gain insight into the current state of affairs and occasionally provides a framework for alternative societal structures (Freeman, 2014).

When a researcher decides to apply DLT to an area of law or a legal institution, it is expected that the law’s neutrality, patriarchy and audism should be challenged. Being “neutral” is generally taken to mean “not taking sides in a controversy, dispute, disagreement, etc.; not inclining toward any party, view, etc.” (Oxford English Dictionary, n.d.). DLT, as does feminist legal theory, critical race theory and critical disability theory, challenges the idea that the law is neutral, given that it tends to be “benchmark men”—men who are White, heterosexual and able-bodied (Thornton, 1997)—who create the law, and that it is impossible for these decision-makers not to allow their personal characteristics and dominant discourse influence their decisions. These “benchmark men” also operate or have operated with a historical backdrop of oppression and colonialism. That confirms that the law cannot be neutral nor value free as it does not eliminate personal biases, beliefs, emotional and personal involvement. In other words, it is not possible for lawmakers to conduct detached, objective inquiry (Silbey & Sarat, 1987).

In the present context, I assert that the law is also “hearing,” that is, not deaf, given that the people who created laws are hearing. Therefore, the law matches a hearing reality as the majority consists of people who are hearing, whereby deaf people are in the minority. Bryan and Emery (2014) refer to such law as “hearing-subjective” as they focus on hearing people and reflect the hearing world. This is a particularly pertinent argument as in the realm of politics and public affairs, there exists a pronounced deficiency of deaf individuals serving as representatives for their communities at both local and national level, exacerbated by the insufficient support from political parties to address existing obstacles and the financial burdens associated with candidacy for elected positions (Evans & Reher, 2022; UK Parliament, 2016). From the electorate’s perspective, the overarching lack of access is well-documented. Deaf individuals frequently encounter limitations with political information (Valentine & Skelton, 2009), and a survey revealed that deaf citizens felt they were unable to exert influence over the political landscape of their nation and did not fully comprehend the platforms of their national parties (Pabsch, 2014). These barriers to participation in lawmaking processes mean that deaf individuals are often relegated to the role of “others” in legislation and policy, unable to fully engage in high-level discussions that shape their lives.

In addition to issues of audism, oppression and colonialism, society is patriarchal towards deaf people. Smith (2010) argues that what feminist legal theories have in common is an opposition to patriarchal ideas that dominate society in general, and legal systems in particular. Law relating to deaf people is traditionally created to “look after” or “take care of” deaf people to live their lives as best as they can, a practice that begins with the assumption that deaf people need “help.” The legal roots of the treatment of deaf people still lie in a state that invokes charity, where a society wishes to do good or to reassure themselves of their moral conscience (Lane, 1992). Mor (2006) argues that this is due to the structure of the market economy and negative social attitudes, and the law in one way or another significantly generates some form of disablement as a result. Hendriks and Degener (1994) go further and argue that there is an inclination to use legislation as an instrument to separate people. To elaborate, Minow (1990) characterises legislation as a means of dividing society into “different” groups by establishing categories and drawing boundaries between normal and abnormal, competent and incompetent, able-bodied and disabled. As a result, mere legislation is unlikely to change deep-rooted attitudes in society (Hendriks & Degener, 1994).

Lane (1992) posits that there are four kinds of evidence that society has paternalistic tendencies towards deaf people. The first is the overlapping traits of benefactors and beneficiaries, whereby he argues that hearing culture has been imposed on deaf people, much like colonialism, where the coloniser’s culture is imposed on the colonised without their consent. This is evident in the medicalisation of deafness, where deafness is often treated as a medical condition that requires intervention, rather than acknowledging it as a natural variation in human experience, or in forced oralism, the practice of teaching deaf children to speak and lipread, despite the availability of sign language (Blind, &c. Commission, 1889). Secondly, Lane identifies the tendency of paternalistic authority to describe deaf people differently from how they would describe themselves. This is reflected in the portrayal of deaf people in the media, often as helpless victims or as incapable of living independent lives, reinforcing negative stereotypes and limiting their opportunities (Lane, 1992).

In the UK, the main example of this can be found in the Equality Act (2010), which defines a disability in section 6(1) as a physical or mental impairment which has a substantial and long-term adverse effect on a person’s ability to carry out normal day-to-day activities. Lane (1992) also highlights the “White Man’s Burden test,” where benefactors consider themselves to have the burden of supplying deaf people with language, culture, and institutions. This is exemplified in the overemphasis on cochlear implants, devices that aid with processing sound, often promoted as a “cure” for deafness, without fully considering individual needs and preferences. For instance, the dominance of spoken language in deaf education, even when sign language is more effective for many deaf children, reflects the paternalistic assumption that deaf people need to be “fixed” in order to fit into a hearing world (Lane, 1992). Finally, Lane (1992) underscores the economic motivations underlying paternalistic relations, with deaf people becoming consumers of deaf-related technology, such as hearing aids and cochlear implants, the dispensing of which are regulated by the Health Professions Order (2001) and the Health Professions (Parts of and Entries in the Register) Order of Council (2003), dominated by hearing companies that may not prioritise the needs of deaf people. For example, the lobbying power of the hearing aid and cochlear implant industry can influence policy decisions to favour these technologies over alternative approaches, such as sign language (Lane, 1992).

## Applying deaf legal theory

Applying a DLT lens will enable scholars to understand the implications law has on deaf people and their lives. In devising a model with which to apply DLT, inspiration is provided by similar models for feminist legal theory, critical race theory and critical disability theory and for Dis/ability Critical Race Studies (DisCrit) (Annamma et al., 2013). Wishik (1985) proposed a framework of inquiry for feminist jurisprudence which asks the following questions: what women’s experiences are addressed by an area of law; what assumptions or descriptions of experience the law makes; what area of distortion or denial is created; what reforms have been proposed, and how they will affect women both practically and ideologically; how women’s situation would look in an ideal world; and how do we get there from here. These questions could quite appropriately be applied to the experience of deaf people; with the word “woman” or “women” replaced by the word “deaf” or “deaf people.”

In terms of critical race theory, Rollock and Gillborn (2011) outline five central themes: the centrality of racism in terms of its entrenchment in the social order so that it is viewed as normal or natural, White supremacy in how Whites overwhelmingly control power and resources across institutions and social settings, the voices of people with colour in stories or counter-narratives, interest convergence whereby if equality is perceived as being in the interest of White people, advances can be achieved, and intersectionality, which recognises that no person has a single, simplistic unitary identity. While critical race theory offers valuable tools for analysing systemic inequalities, its application to White deaf communities must be approached with caution. As Moges (2020) and Friedner (2017) emphasise, intersectionality is crucial for understanding how race, colonialism, and other factors intersect with deaf identities. Uncritical applications risk oversimplifying these complex dynamics.

Critical disability theory also provides a conceptual framework to understand the relationship between impairment, disability and society and to inject disability interests into all policy arenas. There are seven elements of critical disability theory: the social model of disability, multidimensionality, valuing diversity, rights, voices of disability, language, and transformative politics (Hosking, 2008). Likewise, DisCrit focuses on how the forces of racism and ableism uphold notions of normalcy, intersectionality, social constructions of race and ability, the voices of marginalised populations, the legal and historical aspects of dis/ability and race, Whiteness and ability as property, interest convergence and activism and resistance (Annamma et al., 2013).

These frameworks can be extended to DLT, and to determine the extent of “hearing-subjectiveness” (Bryan & Emery, 2014) in any legal system or area of law, the following tenets should be considered:

(1) The frame of understanding within society that shapes the understanding of deaf people.
(2) The assumptions made regarding deaf people in the shaping of the law.
(3) The participation of deaf people in the shaping of the law and/or policy.
(4) The extent society has imposed its cultural order on deaf people in relation to the law.
(5) The application of the law to deaf people.
(6) The impact the law has on deaf people and their allies.
(7) Whether deaf people experience further oppression or afforded rights.
(8) Lessons about how the law can and should bring deaf people within its purview.

I shall now look at each of those in turn, providing further discussion and examples where appropriate.

### The frame of understanding within society that shapes the understanding of deaf people

The relationship between deaf people and the social groups they encounter throughout their lives, on a personal or professional level, be they (hearing) families or friend groups, educationalists, work colleagues, health and medical professionals, or charity representatives, determine the frame of understanding within which these relationships are formed and developed. For example, while a deaf individual could be part of the Deaf community, when they encounter a health or medical professional, that professional will view the patient as primarily an individual with impairment of hearing. An educationalist may frame them in a similar way, given the influence of the health and medical profession on deaf education (Wilks & O’Neill, 2022), whereas charities may frame deaf people as objects of charity who need “help” and “looking after.”

Social construction is defined as “the concept that people shape or ‘construct’ their knowledge and understanding such that it may be difficult to separate objective knowledge from subjective ideas” (Matthews, 2014). In the context of gender, Cotterrell (2003) explains that gender differences exist as systematic patterns of domination expressed in, for example, work conditions and opportunities, domestic institutions, education, culture, politics and law. So, when we describe the frame of understanding within this context, we consider how deaf people are *socially constructed*, that is, how people’s knowledge and understanding of deaf people has been shaped or constructed according to society’s subjective ideas of what it means to be deaf. The best illustration of such ideas is the language used to describe deaf people. Language can influence the frame of understanding, particularly in relation to disabled people, which can include both the words used to describe or label disabled people and the words and images used to portray disability. This is because language carries with it ideological implications which are transparent, and it is inherently political with words and images used to portray disabled people having a direct effect on social attitudes towards this group of people (Hosking, 2008). Deaf people are sometimes labelled as “hearing impaired,” a term that tends to emphasise a medical perspective, framing deafness as a condition in need of correction. Alternatively, they may be referred to as “deaf and dumb,” “retarded” or “delayed,” historically outdated, offensive and ableist terms that stigmatise deaf people and people with learning disabilities (Stratiy, 2001). However, it is important to recognise and appreciate the richness of Deaf culture, which encompasses a vibrant community with its own language, such as sign language (Ladd, 2003). By embracing a more inclusive language and acknowledging the diverse experiences within the Deaf community, we can move beyond stereotypes and contribute to a more nuanced understanding of deafness.

By way of example, inclusive language can be found in the BSL Act (2022) which refers to “Deaf” people and acknowledges the existence of “Deaf culture, identity, community and history.” In addition, section 9C of the Juries Act (1974) (inserted by section 196 of the Police, Crime, Sentencing and Courts Act (2022)) refers to deaf people and BSL and the BSL (Scotland) Act (British Sign Language (Scotland) Act, 2015) refers to persons who are “deaf and deaf-blind.” The Communications Act (2003) refers to deaf people as “deaf and hard of hearing,” and the Anti-social Behaviour, Crime and Policing Act (2014), Patient Rights (Scotland) Act (2011), Welfare Reform Act (2009) and Youth Justice and Criminal Evidence Act (1999) use the word “deaf.” However, outdated expressions continue to feature in some legislation, such as section 29 of the National Assistance Act (1948) which remains in force but only applies to England uses the phrase “blind, deaf, dumb and crippled persons,” as does section 8 of the Rating (Disabled Persons) Act (1978) but without the word “crippled.” What is particularly striking, however, is that legislation regarding the provision of education for deaf children refers to deaf children as “hearing impaired,” and the Explanatory Note to the Education (Special Educational Needs) (Amendment) Regulations (2024) explains that the Education (Special Educational Needs) Regulations (1983) were amended in order to replace the term “deaf” with the term “hearing impaired.” The Data Protection Act (2018) also refers to deaf people as “hearing impaired,” as does the Social Services and Well-being (Wales) Act (2014) and the Health and Social Care Act (2008). The Equality Act (2010) refers to deaf people as both “hearing-impaired”^3^ and “deaf,”^4^ and section 146 of the Transport Act (2000) goes further and refers to deaf people as “profoundly or severely deaf.”

### The assumptions made regarding deaf people in the shaping of the law

Assumption is distinguishable from the frame of understanding, and it is the end-product of the frame of understanding in society. It is a “sort of limitation or circumscription of the thinking process” and “the technique for finding one’s assumptions would be to examine one’s thinking to try to observe in what ways it is being limited” (Delin et al., 1994). For example, the health and medical profession is influenced by a “medical” perceptual framework (Swain et al., 2003), and perceives being deaf as a medical condition, a deficit, something to be treated or cured, and this leads to the assumption that deaf people are defunct, broken and in need of repair, which is aligned with the medical model of disability.

This is because the framework perpetuates the belief that disabled individuals are biologically or physiologically inferior, emphasising on individual limitations and reinforces a dependency on others. Derogatory labels like “invalid,” “cripple,” “spastic,” “handicapped,” and “retarded” further contribute to the negative perception of disability and disregard the perspectives of disabled individuals (Barton, 1996). Therefore, the effectiveness of the medical model in protecting against disability discrimination is questionable, as it primarily focuses on individuals adapting to societal norms rather than addressing the failure of the social environment to accommodate the needs and aspirations of those with impairments (Schiek et al., 2007).

In addition, charities may see being deaf as an ailment or disadvantage and respond by the assumption that deaf people need help to function in society. To illustrate, the Royal National Institute for Deaf People’s charitable object is:

to promote and encourage the prevention and mitigation of deafness and the better treatment, education, training, employment and welfare of people who are deaf or hard of hearing (which expression applies to all those whose hearing is significantly impaired), and generally to promote, safeguard and protect the welfare of such people (Charity Commission for England and Wales, 2023c)

whereas the Royal Association for Deaf people’s charitable object is “to promote the spiritual, social and general welfare of deaf people” (Charity Commission for England and Wales, 2023b) and the National Deaf Children’s Society aims “to relieve the needs of deaf children and young people” (Charity Commission for England and Wales, 2023a). Thus, social groups will tailor their services in response to the assumptions that derive from their frame of understanding, that is, from the law.

### The participation of deaf people in the shaping of the law and/or policy

Lane (1992) argues that deaf people do not generally have the “chance at self-creating to the best of his or her abilities,” and should be crucial participants in the discussion and agreement concerning the lives of deaf children and adults and the roles of the professions that serve them. Hammel et al. (2008) explain that participation is “a complex, nuanced phenomena” that means something different for different people on different levels and is a both “a means and an end to the expression of personal and collective societal values.” The findings of this particular study revealed that the six core participation values are active and meaningful engagement, choice and control, access and opportunity, personal and societal responsibilities, having an impact and supporting others, and social connection, societal inclusion, and membership, with respect and dignity a critical feature of participation across all themes. Therefore, when applying DLT, one is required to determine the extent of each core participation value in the shaping of the law and/or policy that directly affects deaf people with deaf people themselves.

### The extent society has imposed its cultural order on deaf people in relation to the law

Gordon (1982) presents a compelling argument that legal discourses serve as a means of exerting power and are filled with categories and imagery that subtly justify and legitimise the existing social structure. He further explains that law is just one of many systems people create to navigate the challenges presented by others, and it is through these systems that elites maintain their dominance by defining rights in a way that reinforces social hierarchies. As a result, the social order appears to be natural and inevitable. Delgado and Stefancic (2001) further assert that racism and ableism have become deeply ingrained in our society, to the point where they are seen as normal and natural. This is what DLT attempts to expose in relation to deaf people, and Bryan and Emery (2014) explain that cultural order is usually expressed through the mechanisms of law, and that although deaf people are granted rights and various protections under the law, the dominant group uses this law to reign supreme over deaf people.

Merry (1991) and Ladd (2003) both refer to cultural order in the context of colonialism, explaining that colonialism is a relation between two or more groups of unequal power in which one not only control and rules the other but also endeavours to impose its cultural order onto the subordinate group. Gartrell (1984) defines colonialism as “formal political control” through “a specialised administrative apparatus, with an ideology justifying such control,” that tends to treat the subordinate group as “different and inferior.”. It is clear that there is a relationship between cultural order and colonialism, which is particularly relevant in the deaf context as Ladd (2003) argues that Deaf communities have undergone colonisation. Ladd further argues that, in his examination of Deaf cultures, it was obvious that these cultures were not only directly affected by majority cultures, but that their own cultural patterns had become shaped by both acquiescence to and resistance against that cultural domination. This sums up the extent cultural order can regulate deaf people.

Further, Sherry (2007) critiques how colonial frameworks have imposed Western norms on global deaf communities, marginalising indigenous signed languages and cultural practices. Similarly, Friedner (2017) highlights how colonial legacies shape contemporary discourse on deaf identity and legal systems. These historical power dynamics underscore the need to critically examine the systemic oppression faced by deaf communities. On the other hand, revisionist claims about colonialism suggest that the guilt associated with colonialism as well as political correctness often veil the positive side of the colonial project (Brandon & Sarkar, 2019). Brandon and Sarkar (2019) argue colonialism had beneficial outcomes, imperial rule was actually the “norm” for its time, and decolonisation has on occasion proven to be a “disaster.” The positive outcomes associated with colonising the Deaf community along the same lines could be as follows: society has advocated for accessibility, education, and technological advancements for the Deaf community, and provides financial support by way of social security benefits to avoid poverty and ensure social inclusion for different socio-economic groups.

Therefore, within a DLT context, the question to be asked is what control is exerted over deaf people by law, and what ideology is used to justify such control. For example, social security law extends disability benefit entitlement to deaf people, and deaf people are expected to meet the criteria imposed on claimants in order to qualify pursuant to Part 4 of the Welfare Reform Act (2012), whereby the person’s ability to carry out daily living activities is limited by a physical or mental condition (section 78) or their mobility activities are limited by a physical or mental condition (section 79). The ideology for this law comes from a place of charity, of a desire to help those “less fortunate” (Lane, 1992), and in the process imposes a hearing construct of who the dominant group perceive deaf people should be.

### The application of the law to deaf people (e.g., the relevant legal principles and how they or should be applied to deaf people)?

Doctrinal research is generally regarded as research into the law and legal concepts (Hutchinson & Duncan, 2012), which is by and large the traditional black-letter law approach that will be used in this tenet of DLT. Black-letter law focuses on the authoritative text of statutes, case law, and legal doctrines, emphasising legal rules as they are written and applied (Hutchinson & Duncan, 2012). Kharel (2018) argues that doctrinal legal research remains a traditional and widely used approach in the legal field and although it is now expected that methodologies from other disciplines be used in combination, the doctrinal method remains appropriate for legal research (Nyathi, 2023). Applying the law aims to “reveal the presence of a series of rules based upon a smaller number of general legal principles” (Slater & Mason, 2007), from the collection and analysis of a body of case law, together with any relevant legislation, sometimes from a historical perspective and may include secondary sources such as journal articles or other written commentaries on the case law and legislation (Johns & Dobinson, 2017). These rules may be either directly or indirectly applied to deaf people. For example, if there is legislation or case law pertaining to deaf people, this would be a direct application, but in the case of a dearth of such legislation or case law, then an indirect application can be made which surmises or extrapolates how such law can be applied.

However, as Nyathi (2023) has highlighted, the black-letter approach alone can be insufficient as it ignores the wider social and political context, and therefore can only be properly understood if it is studied in that context (Harris, 1986). Singhal and Malik (2012) also warn that doctrinal research alone is “too theoretical, too technical, uncritical, conservative, trivial and without due consideration of the social, economic and political significance of the legal process.” The tendency of academics to incorporate evidence and methods from other disciplines to doctrinal research has come about due to much debate regarding the constrictive nature of doctrinal research, and as such, the doctrinal methodology is in a period of change and transition (Hutchinson, 2015).

### The impact the law has on deaf people and their allies

As we are seeking to examine the impact of law on deaf people through a DLT lens, it is necessary to carry out a socio-legal approach. However, Mulcahy and Cahill-O’Callaghan (2021) highlight a lack of in-depth engagement with, or development of, debates about epistemology, methodology, and method in relation to socio-legal studies. Socio-legal research initially focused on the “law in society” approach which aimed to examine the law in action and the operation of the legal system, followed by the “law in its social context” approach and then postcolonial and non-Western traditions of thought. It is now considered to be of “eclectic efflorescence,” and the consensus is that there is there is no single or unified understanding of the term “socio” (Feenan, 2013).

With this in mind, Patterson (2010) advances that law and society covers just about everything about law except for legal doctrine and goes as far as to say that the law is connected to every facet of society, giving rise to a discipline that knows no limits or definitively established subject matter. Cownie and Bradney (2017) explain that socio-legal scholars have been accused of producing research that is not particularly intellectually sophisticated and which is atheoretical and descriptive in nature and Cownie (2004) in particular regards such research as methodically unsophisticated with poor quality data and questionable analysis. These concerns arise from the fact that “lawyers and sociologists don’t talk the same language” (Schur, 1968), but can be addressed by ensuring that any socio-legal exposé through a DLT lens is at least married to robust research methodologies from the social sciences or any other relevant discipline, particularly Deaf Studies, in order to explore the impact of the law on deaf people.

### Whether deaf people experience further oppression or afforded rights

There are “five faces of oppression:” exploitation, marginalisation, powerlessness, cultural imperialism and violence (Young, 1990, p. 42). These faces have immediate relevance to deaf people. While there is generally a dearth of research available as to the extent of exploitation and violence towards deaf people (Neille & Penn, 2017), recent research provides compelling evidence that deaf individuals face significantly higher risks of abuse and violence compared to their hearing peers. Studies highlight that deaf children are two or three times more likely to experience sexual abuse, with institutional settings like special schools posing particular risks (Kvam, 2004; Sullivan & Knutson, 2000). Intimate partner violence among deaf adults is also prevalent, compounded by communication barriers and a lack of specialised support services (Mastrocinque et al., 2017). Broader reviews show elevated rates of emotional, physical, and sexual abuse among deaf populations, driven by social stigma, dependency on caregivers, and isolation (Wakeland et al., 2018), and how perceptions and systemic neglect perpetuate vulnerability and hinder reporting (Admire & Ramirez, 2021).

Exploitation is concerned with the structural relation between social groups, whereby social rules about what work is, who does what for whom, how it is compensated, and how these rules manifest themselves is through a systematic process in which the energies of “the have-nots … are expended to maintain and augment the power, status, and wealth of the haves” (p. 50). Marginalisation is considered to be “the most dangerous form of oppression” (p. 53) and refers to the expulsion of people from useful participation in social life and thus potentially subjecting them to severe material deprivation and even extermination. Powerlessness refers to a lack of authority, status and sense of self. Cultural imperialism involves the universalisation of a dominant group’s experience and culture, and its establishment as the norm, involving “the paradox of experiencing oneself as invisible at the same time that one is marked out as different” (p. 60). Finally, violence, refers to systematic violence directed at members of a group simply because they are members of a group. The fourth, cultural imperialism, has parallels with Ladd’s argument that Deaf communities have undergone colonisation (see above). This tenet of DLT requires an examination as to whether deaf people’s experiences of the law in question serves as a new or further instance of the oppression of Deaf communities or affords them additional rights and how effective these may be, and in the process determining the extent to which the frame and assumptions have been or may be changed.

### Lessons about how the law can and should bring deaf people within its purview

Gordon (1982) astutely observes that true adversaries lie within us—the ingrained structures and imagination constraints that restrict us. By realising that the key lies in exploring uncharted territories, meaningful transformation becomes possible. In fact, throughout history, pivotal shifts have often occurred when individuals defy the norms of subjugation, boldly disregarding the seemingly insurmountable barriers to improvement and embracing the power to effect change. Before that can happen, however, lessons must be learnt.

Patterson (2010) suggests that any legal and social research should consider the following questions:

(1) Who declares legal norms and how are these norms selected for recognition?
(2) Do legal actors implement established legal norms?
(3) How do coexisting legal institutions relate to each other?
(4) What impact does law have on individuals and groups in society?
(5) Who invokes legal institutions? Why?
(6) Whose interests are served by the actions of legal institutions?
(7) How do people in society react to the actions of legal institutions?
(8) How much do people know about the law?
(9) What do they think of the law?
(10) Did they seek legal assistance or resort to other mechanisms to deal with the problem? (Gordon, 1982)

By applying a DLT lens we will start answering these questions to examine an area of law or legal system on deaf people. Once accomplished, any researcher who undertakes such an exposé is encouraged to provide suggestions for legal reform with a view to providing solutions to an identified problem.

In the context of feminist legal theory, Penner and Melissaris (2012) urge feminist writers to propose a plan for legal reform, as “pointing out an injustice is the same thing as pointing out something that needs to be rectified” (p. 227). However, the challenge lies in persuading politicians, policymakers, and legislators to implement these changes. Asch (2001) doubts whether such social change is possible, although Freeman (2014) suggests that change could be possible where there is a convergence of interests where the required changes align with the decisionmakers’ own interests, for example, White elites have been found to tolerate or support racial progress for Black individuals when it serves their own self-interest. When proposing reforms, DLT researchers may face the difficulty of considering how such changes can benefit not only deaf individuals but also society at large. This can lead to dilemmas in suggesting reforms: should a strictly neutral approach be enforced, potentially reinforcing existing social inequalities, or should forms of preferential treatment be advocated, which may provide short-term aid but risk perpetuating the perception of weakness or inferiority among the groups receiving such treatment (Bix, 2015)?

Exposing areas of law through a DLT lens can also encourage consciousness-raising, a feminist legal practice. This approach is rooted in the belief that empowering the marginalised to communicate their experiences with one another leads to a heightened awareness of their own circumstances (Penner & Melissaris, 2012). Through this collective understanding, recurring patterns in their shared experiences are recognised, enabling the development of a comprehensive theory on how to combat oppression (Matsuda, 1987). Consequently, any examination of DLT must prioritise creating a space for deaf individuals to share their personal stories and foster a recognition of the shared struggles they face. Any DLT study should therefore where possible allow deaf individuals to enter a deaf space and given an opportunity to share their personal experiences to encourage an awareness of the commonalities of their oppression.

## Conclusions and future directions

It cannot be right to deny deaf people the opportunity to play a role in the public life of communities or nations, and DLT has a significant role to play in exposing systems that prevent, either directly or indirectly, them from doing so. After all, “those who are oppressed in the present world can speak most eloquently of a better one” (Matsuda, 1987, p. 346). It is clear that the current underpinnings of law are based on many incomplete assumptions (Bryan & Emery, 2014), and DLT seeks to expose these assumptions, and it is necessary to do so as a first step toward developing a model of Deaf jurisprudence and a precursor to effecting social change. I have argued that DLT sits firmly within CLS discourse. There is also some alignment between DLT and feminist legal theory.

When the DLT method is applied to a legal system or area of law, the result should be that incomplete assumptions are exposed, and Deaf jurisprudence further expanded. By applying the eight tenets of DLT to any given law or laws, we will establish the frame of understanding, social construction or language used in which that law was made and the assumptions that arose as a result. Deaf people’s participation (or not) in the creation of that law will also be telling in whether the law was created with their perspectives in mind, and ultimately how far the cultural order pervaded. Following an application of the law and an examination of its impact on deaf people, it can be surmised whether oppression has been addressed, maintained or alleviated. This process will allow us to identify the lessons to be learnt about how the law can and should bring deaf people within its purview, with a view to effecting change for the benefit of deaf communities.

In the context of critical race theory, one would generally not expect a White researcher to conduct research through a critical race theory lens, and one would not generally expect a man to conduct feminist legal research (although it has and does happen). The same understanding should be applied to DLT. There is a workaround, however. Bergerson (2003), Arai and Kivel (2009) and Giri (2022) all agree that White, male and hearing researchers should recognise and acknowledge their privilege. They should also centre race, feminism and/or hearing-subjectiveness and see Whiteness as a race, maleness as a gender and hearingness as a state of being to allow them to understand that these are not the neutral base from which all else is judged. In any case, in the context of DLT, it is important to note that, firstly, as a whole the number of deaf researchers pales into insignificance when compared to hearing researchers, and secondly, DLT is a relatively new area of jurisprudence that has not been fully developed. Therefore, it can be argued that it is necessary for hearing researchers to expose areas of law and legal systems to assist the development of such jurisprudence.

In this article, I set out to explore the emerging field of DLT and its potential significance in the broader field of jurisprudence. By placing DLT within the context of existing jurisprudence and highlighting its practical applications, I hope to foster a deeper understanding of this concept. Looking ahead, the next important step involves the collaborative efforts of both deaf and hearing (legal) scholars to identify and address the areas where law and legal systems intersect with the experiences of deaf individuals. By doing so, we can pave the way for a comprehensive Deaf jurisprudence, leaving a legacy for future generations of deaf individuals and deaf communities.

## Footnotes

^1^
For example, section 6(1) of the Equality Act, 2010.

^2^
Such as Duncan Kennedy, Karl Clare, Drucilla Cornell, David Kennedy and Roberto Unger.

^3^
See paragraph [2].

^4^
See paragraphs [9], [10] and [12].
